# Characterization of swine-origin H1N1 canine influenza viruses

**DOI:** 10.1080/22221751.2019.1637284

**Published:** 2019-07-09

**Authors:** Guojun Wang, Luiz Gustavo dos Anjos Borges, Daniel Stadlbauer, Irene Ramos, Maria C. Bermúdez González, Jianqiao He, Yangbao Ding, Zuzhang Wei, Kang Ouyang, Weijian Huang, Viviana Simon, Ana Fernandez-Sesma, Florian Krammer, Martha I. Nelson, Ying Chen, Adolfo García-Sastre

**Affiliations:** a The State Key Laboratory of Reproductive Regulation and Breeding of Grassland Livestock, College of Life Sciences, Inner Mongolia University, Hohhot, People’s Republic of China; b Department of Microbiology, Icahn School of Medicine at Mount Sinai, New York, USA; c Global Health and Emerging Pathogens Institute, Icahn School of Medicine at Mount Sinai, New York, USA; d College of Animal Science and Technology, Guangxi University, Nanning, People’s Republic of China; e Department of Medicine, Division of Infectious Diseases, Icahn School of Medicine at Mount Sinai, New York, USA; f Division of International Epidemiology and Population Studies, Fogarty International Center, National Institutes of Health, Bethesda, USA; g The Tisch Cancer Institute, Icahn School of Medicine at Mount Sinai, New York, USA

**Keywords:** Host switch, reassortant, H1N1, influenza A viruses, canine

## Abstract

Host switch events of influenza A viruses (IAVs) continuously pose a zoonotic threat to humans. In 2013, swine-origin H1N1 IAVs emerged in dogs soon after they were detected in swine in the Guangxi province of China. This host switch was followed by multiple reassortment events between these H1N1 and previously circulating H3N2 canine IAVs (IAVs-C) in dogs. To evaluate the phenotype of these newly identified viruses, we characterized three swine-origin H1N1 IAVs-C and one reassortant H1N1 IAV-C. We found that H1N1 IAVs-C predominantly bound to human-type receptors, efficiently transmitted via direct contact in guinea pigs and replicated in human lung cells. Moreover, the swine-origin H1N1 IAVs-C were lethal in mice and were transmissible by respiratory droplets in guinea pigs. Importantly, sporadic human infections with these viruses have been detected, and preexisting immunity in humans might not be sufficient to prevent infections with these new viruses. Our results show the potential of H1N1 IAVs-C to infect and transmit in humans, suggesting that these viruses should be closely monitored in the future.

## Introduction

Influenza A viruses (IAVs) rapidly evolve in nature due to mutation and reassortment events. First, the replication of IAVs by their error-prone RNA polymerase rapidly generates a diversity of variants [1]. Second, the segmented nature of their genomes allows the exchange of genes when different IAV strains co-infect host cells simultaneously [2]. Both mutations and reassortment enable viruses to generate new variants, facilitating the adaptation to novel hosts [3–5], the evasion of pre-existing immunity by antigenic drift (mutation) [6] and antigenic shift (reassortment) [7], or the emergence of drug-resistance virus strains [8,9].

The continuous emergence of different IAVs in novel mammalian hosts persistently poses a threat to humans [5,10–14]. It was only 20 years ago that dogs were found to be suitable hosts for IAV in nature. The first detected subtype to emerge in canines was H3N8 IAV-C, which was introduced from horses to dogs approximately in the early 2000s in Florida, United States [3]. An additional introduction of H3N2 IAVs-C from avian species into dogs in Asia occurred in the early 2000s and was first detected in 2006 [15]. Dogs then emerged as an important IAV host, after these two lineages resulted in stable host switches into canines. Over time, four reports of self-limited infections with other avian IAVs in dogs were published, including one with H5N1 [16], one with H5N2 [17], one with H6N1 [18], and one with H9N2 [19] viruses. In addition, four cases of human IAV infections also have been reported in dogs, including two with H1N1/2009 pandemic [20] and two with H3N2 [12] IAVs. In 2015 the Asian canine H3N2 virus was introduced into dogs in the United States [21]. Currently, both H3N8 and H3N2 canine influenza vaccines are approved for use in dogs in U.S.A..

In 2013, novel swine triple reassortants H1N1 IAV (H1N1 IAVs-S) with 2009 pandemic H1N1 segments (PB2, PB1, PA, and NP), Eurasian avian-like H1N1 swine segments (HA, NA, and M) and North American triple-reassortant swine (NS) lineages were first reported in pigs in Tianjin, China [22]. Now, these H1N1 IAVs-S have become predominant in the pig population in Southern China [14]. Human infections with these H1N1 IAVs-S have been detected [23,24], albeit no events of human-to-human transmission have been reported. Notably, we found that these H1N1 IAVs-S host switched from swine to canine in Southern China [13]. Moreover, we also identified novel reassortants between swine-origin H1N1 and H3N2 IAVs-C in dogs [13], increasing the genotypic diversity of IAVs circulating in dogs. However, none of these novel H1N1 IAVs-C have been phenotypically characterized so far. Here, we assess the biological properties and ability to replicate in human cells, induce disease in mice and transmit in guinea pigs of three swine-origin H1N1 IAVs-C and one reassortant H1N1 IAV-C isolated from pet dogs presenting with respiratory symptoms at veterinary clinics in China [13].

## Materials and methods

*Viruses*. Influenza A/canine/Guangxi/WZ11/2013 (H1N1) [WZ11], A/canine/Guangxi/LZ56/2015 (H1N1) [LZ56], A/canine/Guangxi/WZ2/2013 (H1N1) [WZ2], and A/canine/Guangxi/LZ36/2015 (H1N1) [LZ36] viruses were isolated and grown in MDCK cells from swabs collected from dogs with influenza-like symptoms in Southern China [13]. Influenza A/Bethesda/P0055/2015 (H3N2) [Beth15], A/California/04/2009 (H1N1) [Cal09] [25], A/canine/Illinois/41915/2015 (H3N2) [Illi15] [26], A/New York/08-1326/2008 (H1N1) [NY08] [27], and a low pathogenic version (HALO) A/Viet Nam/1203/2004 (H5N1) [Viet04] [28] viruses were used as controls.

*Mouse studies*. Groups of six Six- to eight-week-old female BALB/c mice (Jackson Laboratory, Bar Harbor, ME)wereintranasally (i.n.) infected with 10^6^ plaque-forming unite (PFU) of viruses in a volume of 50 μL, three mice were euthanized on 3 and 5 days post-inoculation (d.p.i.), respectively. Lung and nasal turbinates were collected to determine virus titers by plaque assay on MDCK cells.

Groups of five mice were inoculated i.n. with 10^2^, 10^3^, 10^4^, 10^5^, or 10^6^ PFU of virus. Mice were monitored daily for clinical signs of illness and weight loss. Upon reaching 75% of initial body weight, animals were humanely euthanized with carbon dioxide (CO_2_).

*Guinea pig studies*. To investigate the transmissibility of IAVs-C, groups of three five- to six-week-old female Hartley strain guinea pigs (Charles River Laboratory, Kingston, NY) were inoculated i.n. with 10^6^ PFU of virus in a 300 μL volume per guinea pig and housed in a cage placed inside an isolator.

For direct transmission, three naïve animals were introduced into the same cage at 24 h post-infection (h.p.i.) later [11]. For the airborne transmission, three naïve animals were placed in an adjacent cage (4 cm away) at 24 h.p.i., as previously described [27,29]. Nasal washes were collected at 2-day intervals, beginning on two d.p.i. (one day post-exposure) and titrated on MDCK cells.

*Supplemental materials and methods*. Supplementary for this article have been attached for reviewing.

## Results

### Receptor-binding specificity of novel H1N1 IAVs-C

It is generally accepted that receptor-binding preference to the human-type receptors is a requirement for optimal replication in the human upper respiratory tissues and for human to human transmission. For H1 HAs, 190D and 225E are known to allow efficient binding to human-type (*α*-2,6- linked sialic acids) (SA*α*2,6) receptors, and E190D is likely the minimal change needed enabling the shift from binding to avian type (*α*-2,3- linked sialic acids) (SA*α*2,3) receptors to binding to human-type receptors [30]. Among the four H1N1 IAVs-C we used in this study, all of the viruses contain HA 190D, and three out of four strains bear HA 225E (Table 1), suggesting that H1N1 IAVs-C might bind to SA*α*2,6 receptors.

**Table 1.** Genotype and key molecular features of H1N1 IAVs-C.

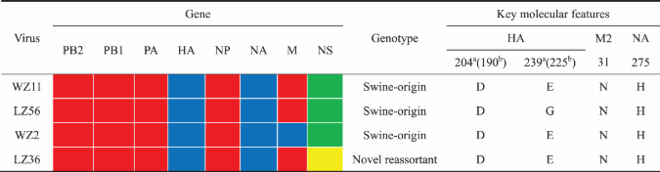													

**Figure 1.**
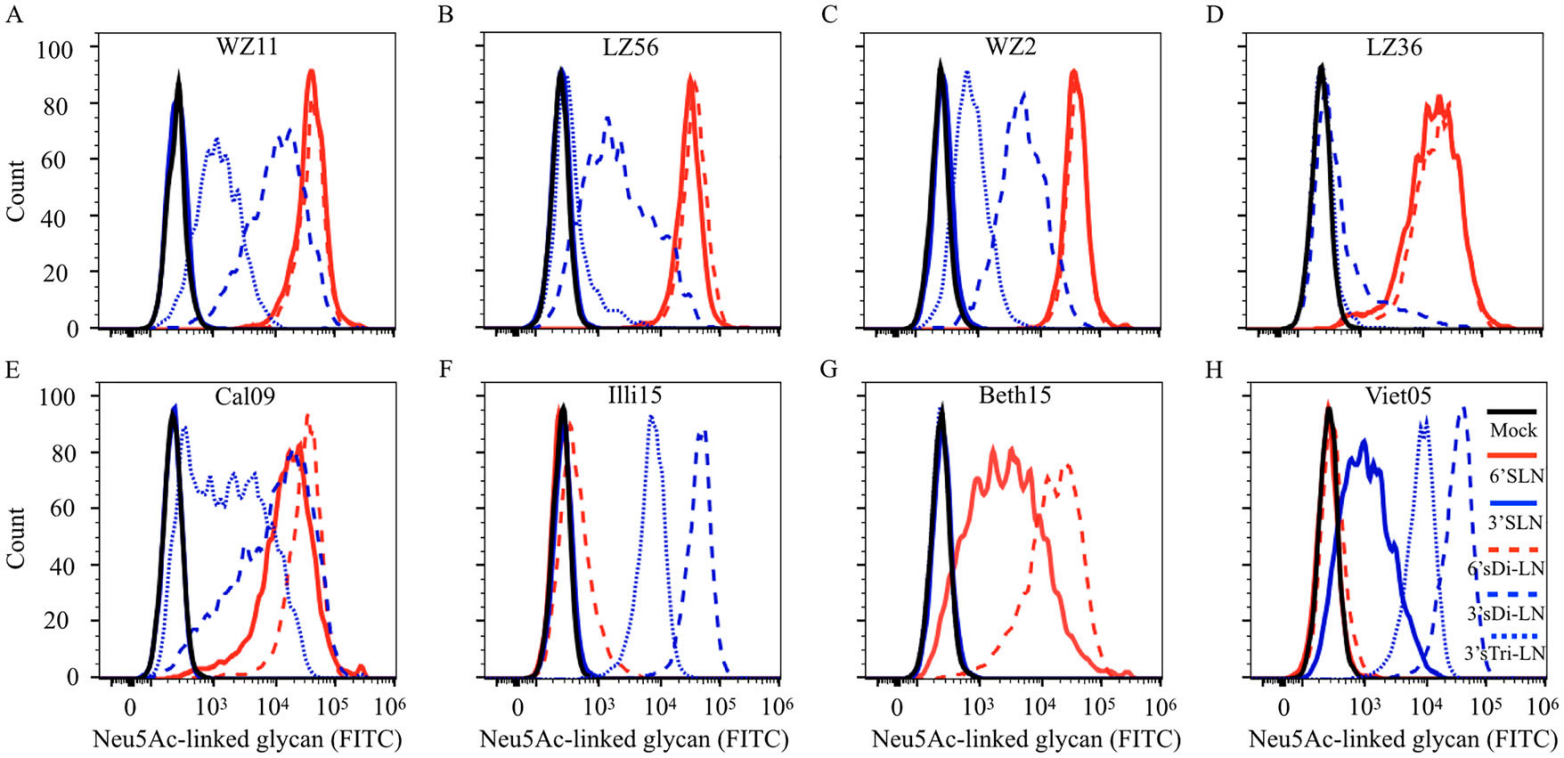
Receptor-binding specificity of H1N1 IAVs-C using a flow cytometry-based binding assay. The binding of virus to five different Neu5Ac-linked glycans was tested: (A) WZ11, (B) LZ56, (C) WZ2, (D) LZ36, (E) Cal09, (F) Illi15, (G) Beth15, and (H) Viet05. E, F, G, and H were included in the analysis for comparison and as controls. *α*-2,3 glycans are in blue and *α*-2,6 glycans are in red. Structure of glycans is provided in Figure S1A.

We tested the receptor-binding specificity of four strains of H1N1 IAVs-C by using a flow cytometry-based assay with five different glycans as described in Figure S1 and in a previous study [31]. As representative strains of H1N1 IAVs-C, we used WZ11, LZ56, WZ2 and LZ36. WZ11 and LZ56 virus strains have a similar genotype, and are derived from triple reassortant H1N1 IAVs-S. WZ2 is also derived from a triple reassortant H1N1 IAV-S, but its M gene has different phylogenetic origin than the WZ11 and LZ56 viruses. Finally, LZ36 has a genotype similar to the WZ11 and LZ56 viruses, except that its NS gene has reassorted and is of H3N2 IAV-C origin (Table 1). Influenza Cal09, Illi15, Beth15, and Viet04 viruses were included for comparison. The four H1N1 IAVs-C and Cal09 bound to SA*α*2,6 with high affinity and to SA*α*2,3 with very low-to-high affinity (Figure 1A–E); Illi15 bound to SA*α*2,3 with high affinity and to SA*α*2,6 with very low affinity (Figure 1F); Beth15 bound only to SA*α*2,6 (Figure 1G); and Viet04 bound only to SA*α*2,3 (Figure 1H). These results indicate that the recent H1N1 IAVs-C preferentially bind to human-type receptors and to a lower degree to the avian-type receptor.

### Replication and virulence of the novel H1N1 IAVs-C in mice

We next investigated the replication and pathogenicity of the four representative IAVs-C in mice. Previous studies have reported that the influenza H1N1/2009 pandemic virus internal genes, especially the PA gene, could enhance the virulence of reassortant viruses [14,32]. In this study, all H1N1 IAVs-C used have a PA gene derived from H1N1/2009 pandemic virus (Table 1).

**Figure 2.**
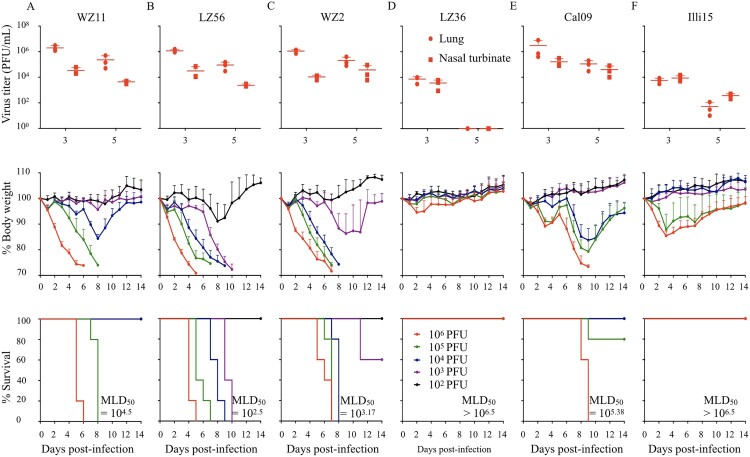
Virulence and replication of H1N1 IAVs-C in mice. To test the viral replication, mice in each group were infected intranasally (i.n.) with 10^6^ PFU: (A) WZ11, (B) LZ56, (C) WZ2, (D) LZ36, (E) Cal05, or (F) Illi15. Virus titers (upper panels) of lung and nasal turbinate on 3 and 5 days post-infection (d.p.i.) were shown as the mean titer of three mice. Five mice per group were i.n. inoculated with 10^2^, 10^3^, 10^4^, 10^5^, or 10^6^ PFU of infected virus. Body weight (middle panels) and survival (lower panels) and were monitored daily. PFU, plaque-forming units; MLD_50_, 50% mouse-lethal dose.

Groups of six BALB/c mice were inoculated with 10^6^ PFU of virus. On day 3 and 5 post-infection, virus titers in the lungs and nasal turbinates of mice were determined. We found that WZ11, LZ56, and WZ2 viruses replicated as efficiently as Cal09 in lungs and nasal turbinates of mice on 3 and 5 d.p.i. (Figure 2A–C and E upper panel). However, the LZ36 virus containing an NS gene derived by reassortment from IAV-C H3N2 replicated more than hundred times less efficiently than the other three H1N1 IAVs-C in mice lungs on 3 d.p.i.; and no infectious viruses were detected in LZ36 virus- infected mice on 5 d.p.i. (Figure 2D and F lower panel).

To investigate the virulence of these H1N1 IAVs-C in mice, we determined their 50% mouse-lethal dose (MLD_50_). We infected groups of five BALB/c mice i.n. with 10-fold serial dilutions of each virus (from 10^6^ to 10^2^ PFU per mouse). Weight and health status were monitored daily for 14 days post-infection. The MLD_50_ values of three swine-origin viruses (WZ11, LZ56 and WZ2) were 10^4.5^,10^2.5^, and 10^3.17^ PFU, respectively, which is more lethal than the human pandemic Cal09 virus (MLD_50_ = 10^5.38^ PFU) (Figure 2A–C and E lower panel). However, the LZ36 virus was not lethal in mice (MLD_50 _> 10^6.5^ PFU), similar to the prototypical canine H3N2 virus (Illi15) (Figure 2D and F lower panel).

These results indicate that swine-origin H1N1 IAVs-C are lethal in mice. In contrast, the swine/canine reassortant virus LZ36 could also replicate although to lower extent and is avirulent in mice.

### Direct contact transmission and aerosol droplet transmission of H1N1 IAVs-C in Guinea pigs

A fundamental prerequisite for an influenza pandemic is that the virus becomes highly transmissible in humans. Numerous studies have reported that the internal genes from H1N1/2009 pandemic are a critical factor to promote aerosol transmissibility for reassortant viruses [32,33]. Since guinea pigs have both human and avian types of airway receptor and our H1N1 IAVs-C bind both SA*α*2,3 and SA*α*2,6 receptors, in order to investigate the transmissibility of IAVs-C in a mammal different from the natural host (dogs), we used the guinea pig animal model for influenza virus transmission.

**Figure 3.**
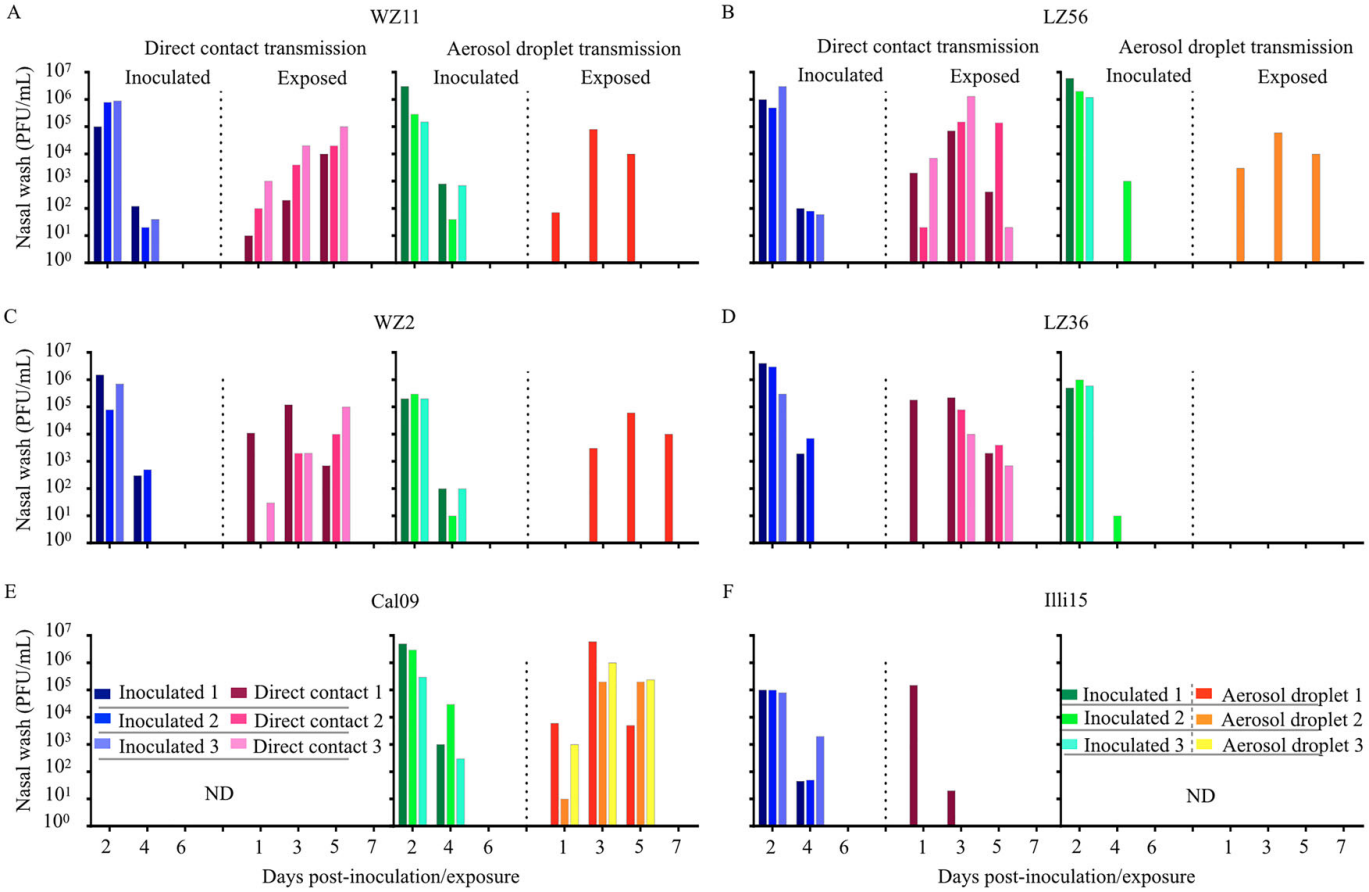
Direct contact transmission and aerosol droplet transmission of H1N1 IAVs-C in guinea pigs. Groups of three guinea pigs were inoculated i.n. with 10^6^ PFU of tested viruses: (A) WZ11, (B) LZ56, (C) WZ2, (D) LZ36, (E) Cal09, or (F) Illi15. Three naïve guinea pigs were each placed in the same cage for direct contact transmission or in the adjacent cage for aerosol droplet transmission at 24 h post-inoculation (h.p.i.). Nasal washes were collected every 2 days from all animals beginning on 2 d.p.i. (one day post-exposure) for the detection of virus shedding. Each colour bar represents the virus titer from an individual animal. ND, not done.

We first examined the direct contact transmissibility of the four H1N1 IAVs-C as compared to a US canine H3N2 virus (Illi15) in guinea pigs. All infected guinea pigs secreted virus after infection (Figure 3). All four H1N1 IAVs-C were able to transmit to all the direct contact guinea pigs (Figure 3A–D), which shed virus and cleared the infection in a similar fashion for all viruses. However, we only detected the virus in one of three direct contact animals of the influenza Illi15 virus-infected group (Figure 3F).

To assess aerosol droplet transmissibility, we set up three transmission pairs, each comprising a naïve guinea pig housed adjacent to an infected guinea pig on 1 d.p.i., as described previously [29]. We compared the four H1N1 IAVs-C with the human transmissible influenza Cal09pandemic virus. Viruses were detected in all of inoculated animals (Figure 3). Cal09 virus was transmitted to all the exposed animals (Figure 3E). Differences were seen, however, in the aerosol droplet transmission between guinea pigs infected with/exposed to the four H1N1 IAVs-C. The three swine-origin H1N1 viruses were able to transmit to one out of three exposed animals by aerosol transmission (Figure 3A–C). The reassortant LZ36 virus failed to transmit to guinea pigs via respiratory droplets (Figure 3D). Additionally, all animals which secreted viruses also seroconverted (Table S2).

Together, these findings demonstrate that the swine-origin H1N1 IAVs-C are highly transmissible by direct contact and with the exception of the reassortant LZ36 virus, are able to transmit, albeit less efficiently than a human H1N1 virus, via respiratory droplets among guinea pigs.

### HI profiles and NI profiles for adult human sera against H1N1 IAVs-C

The emergence and transition to the pandemic status of the swine origin influenza H1N1/2009 virus illustrates the potential for animal H1N1 viruses to become adapted to transmit in humans and cause a pandemic after decades of circulating among animals.

**Figure 4.**
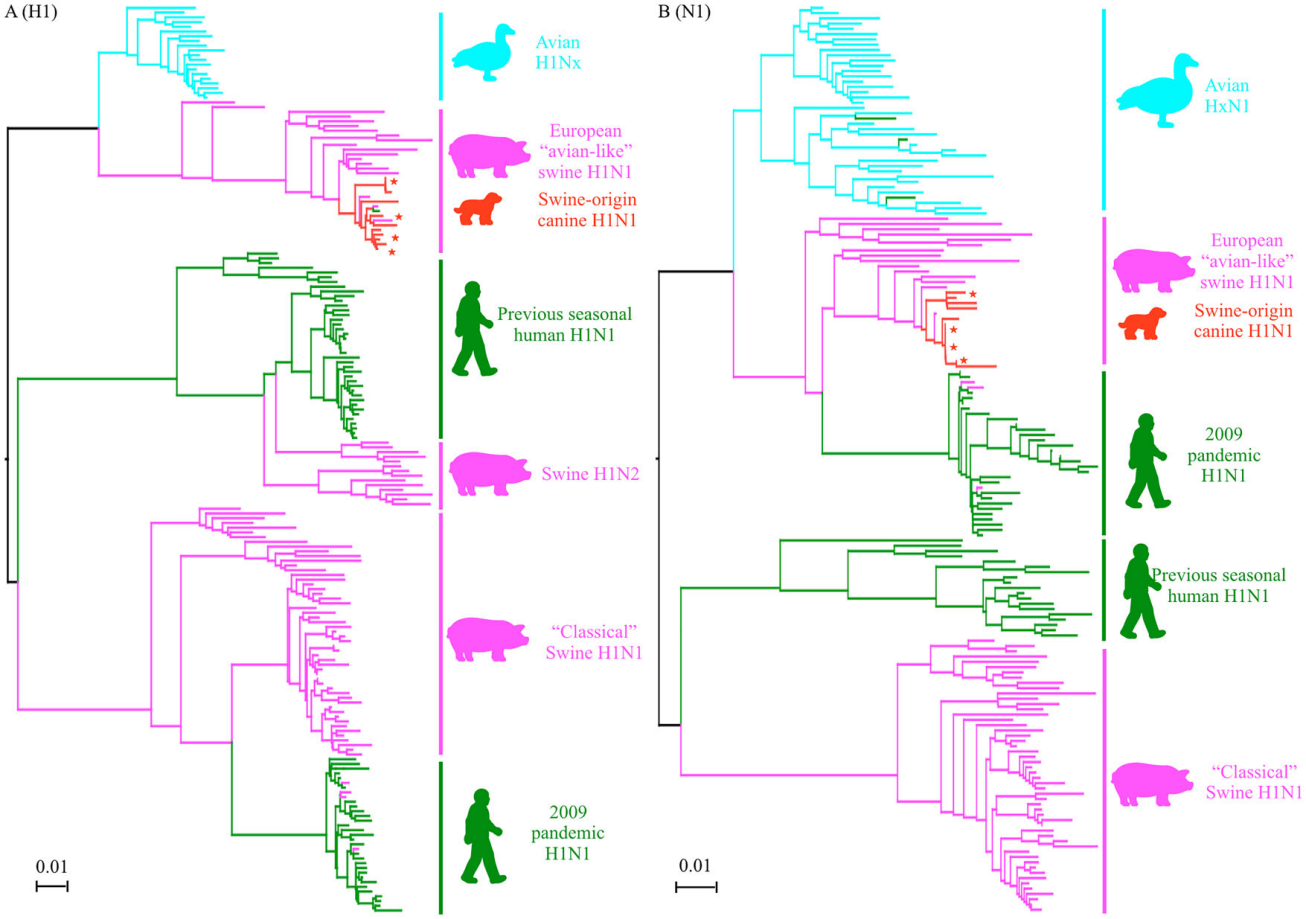
Phylogenetic trees of the HA (A) gene of the H1Nx and NA (B) gene of HxN1 IAVs. Representative H1 and N1 sequences were downloaded from the Influenza Virus Resource at NCBI’s GenBank. Alignments and phylogenetic trees were generated using MAFFT. The trees were based on amino acid sequence (open reading frame) of HA and NA. Avian isolates were in blue; swine isolates were in pink; human isolates were in green; canine isolates were in red. Red star indicates virus used in this study.

Most humans have not been exposed to the HA and NA of H1N1 IAVs-C, which phylogenetically are very different from current and previously circulating human H1N1 viruses (Figure 4). To assess the level of preexisting immunity in humans to H1N1 IAVs-C, we measured the antibody titers to the H1N1 IAVs-C in adult humans by HI and NI assay. We also used the H1N1/2009 pandemic and canine H3N2 viruses for comparison (demographics of the 36 donors provided in Table S3).

**Figure 5.**
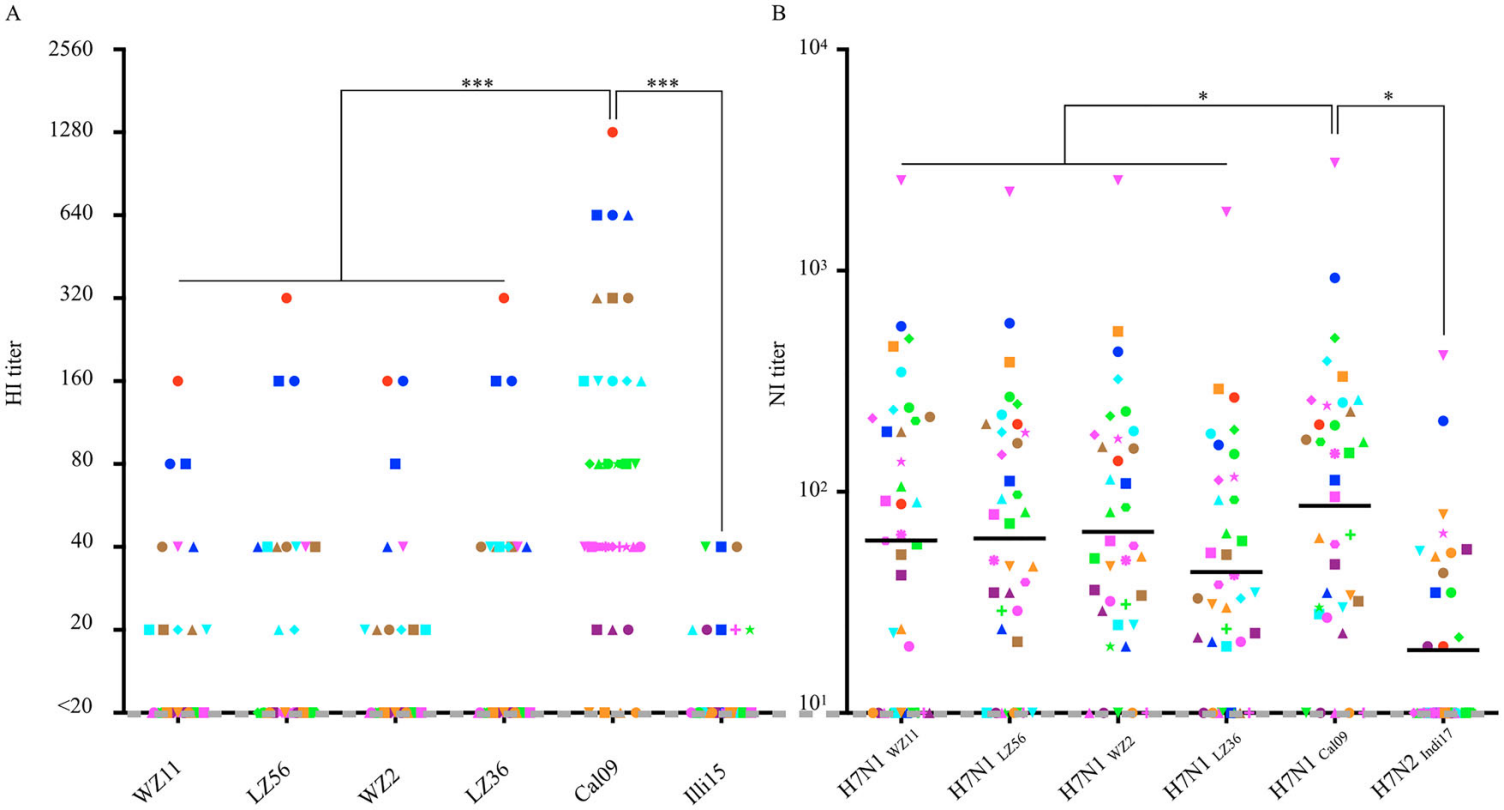
HI profiles and NI profiles for adult human against H1N1 IAVs-C. (A) HI titers of adult human plasma samples (*n* = 36) measured against H1N1 IAVs-C (B) NI titers of plasma against the NA of H1N1 IAVs-C. One-way analysis of variance (ANOVA) was used to determine whether there were any statistical significance between the HI titers and NI titers of the canine IAVs to Cal09 data set (*p **≤ **0.05, ***p **≤ **0.001). The same point indicated by its colour and shape represents the same donor. Bars represent the geometric mean. The dashed line indicates the starting serum dilutions used in both HI and NI assays. HI, hemagglutination inhibition; NI, neuraminidase inhibition.

Serum HI titers of ≥40 are associated with a 50% reduction in risk of infection or disease with human influenza viruses [34]. We found that 16.7%, 27.8%, 13.9%, 30.5% 80.1%, and 8.3% of donors had HI antibody titers ≥40 to WZ11, LZ56, WZ2, LZ36, Cal09, and Illi15 viruses, respectively; and that the HI titers of human plasma against Cal09 were significantly higher than other canine viruses (Figure 5A).

Antibodies against the second major surface glycoprotein, the neuraminidase (NA), affords broad protection from influenza virus infection in animal models and humans [35,36]. We measured functional NA antibody titers against all the NAs using an enzyme-linked lectin assay (ELLA) to determine neuraminidase inhibition (NI) titers. These NAs included N1 from WZ11, LZ56, WZ2, or LZ36 viruses, N2 NA from influenza Indi17 virus, and N1 NA from influenza Cal09 virus (as an example of a circulating strain that these donors might have been previously infected or vaccinated with). To minimize anti-HA head antibody-based interference, H7Nx reassortants containing the NA of interest were employed to test the NI titers [36]. Here, we found that NI titers in humans against the NA of influenza Cal09 virus were significantly higher than against both canine H1N1 and canine H3N2 NAs (Figure 5B).

These results indicate that a limited proportion of adults have cross-reactive antibodies against emerging canine H1N1 but that the preexisting immunity in the human population might not be sufficient to prevent and curb the spread of these strains, if they become transmissible in humans.

### Viral sensitivity to M2 and NA inhibitors in vitro

Currently, three classes of anti-influenza drugs, M2 ion-channel inhibitors, neuraminidase inhibitors, and cap-dependent Endonuclease inhibitor are available for prevention and treatment of influenza A infections [37,38]. The S31N substitution in the M2 ion-channel is associated with high-level resistance to adamantane antiviral drugs, and the H275Y substitution in the N1 NA protein is known to confer high level resistance to oseltamivir antiviral drugs [8,39].

Our H1N1 IAVs-C bear 31N in the M2 gene and 275H in the NA gene (Table 1). To assess whether current anti-influenza drugs are effective against H1N1 IAVs-C, we determined the in vitro half inhibitory concentration (IC_50_) of two inhibitors (amantadine and oseltamivir) against these viruses by plaque reduction assay. Cal09, Illi15, and A/NY/08-1326 (H1N1) were used as controls. The IC_50_ for amantadine was more than 100 µmol/L, whereas those for oseltamivir ranged from 0.024 to 0.28 µmol/Lol/L (Table 2). As a control for amantadine sensitivity, Illi15 and A/NY/08-1326 (H1N1) were used which had the IC_50_ values of 1.78 and 2.79 µmol/Lol/L, respectively (Table 2). As the control of oseltamivir resistance, A/NY/08-1326 (H1N1) was tested which had an IC_50_ greater than 400 µmol/L (Table 2).

**Table 2.** Virus sensitivity to NA and M2 inhibitors *in vitro*.

Virus	Subtype	Amino acid in M2	IC_50_ (µmol/L) amantadine	M2 susceptibility	Amino acid in NA (N2 numbering)	IC_50_ (µmol/L) oseltamivir	NA susceptibility
WZ11	H1N1	31N	>100	Resistant	274H	0.024	Sensitive
LZ56	H1N1	31N	>100	Resistant	274H	0.155 ± 0.092	Sensitive
WZ2	H1N1	31N	>100	Resistant	274H	0.145 ± 0.078	Sensitive
LZ36	H1N1	31N	>100	Resistant	274H	0.28 ± 0.297	Sensitive
Cal09	H1N1	31N	>100	Resistant	274H	0.445 ± 0.219	Sensitive
Illi15	H3N2	31S	1.78 ± 0.212	Sensitive	59E,119E, and 222I	0.084	Sensitive
NY08	H1N1	31S	2.79 ± 2.404	Sensitive	274Y	>400	Resistant

The results suggest that all the H1N1 IAVs-C tested are resistant to amantadine and were sensitive to oseltamivir. Although not tested, these viruses are also likely to be susceptible to the recently approved endonuclease inhibitor baloxavir, as they do not have an I38T substitution in their PAs associated with resistance [40,41].

### Growth properties of H1N1 IAVs-C in differentiated HTBE cells and MDCK cells

**Figure 6.**
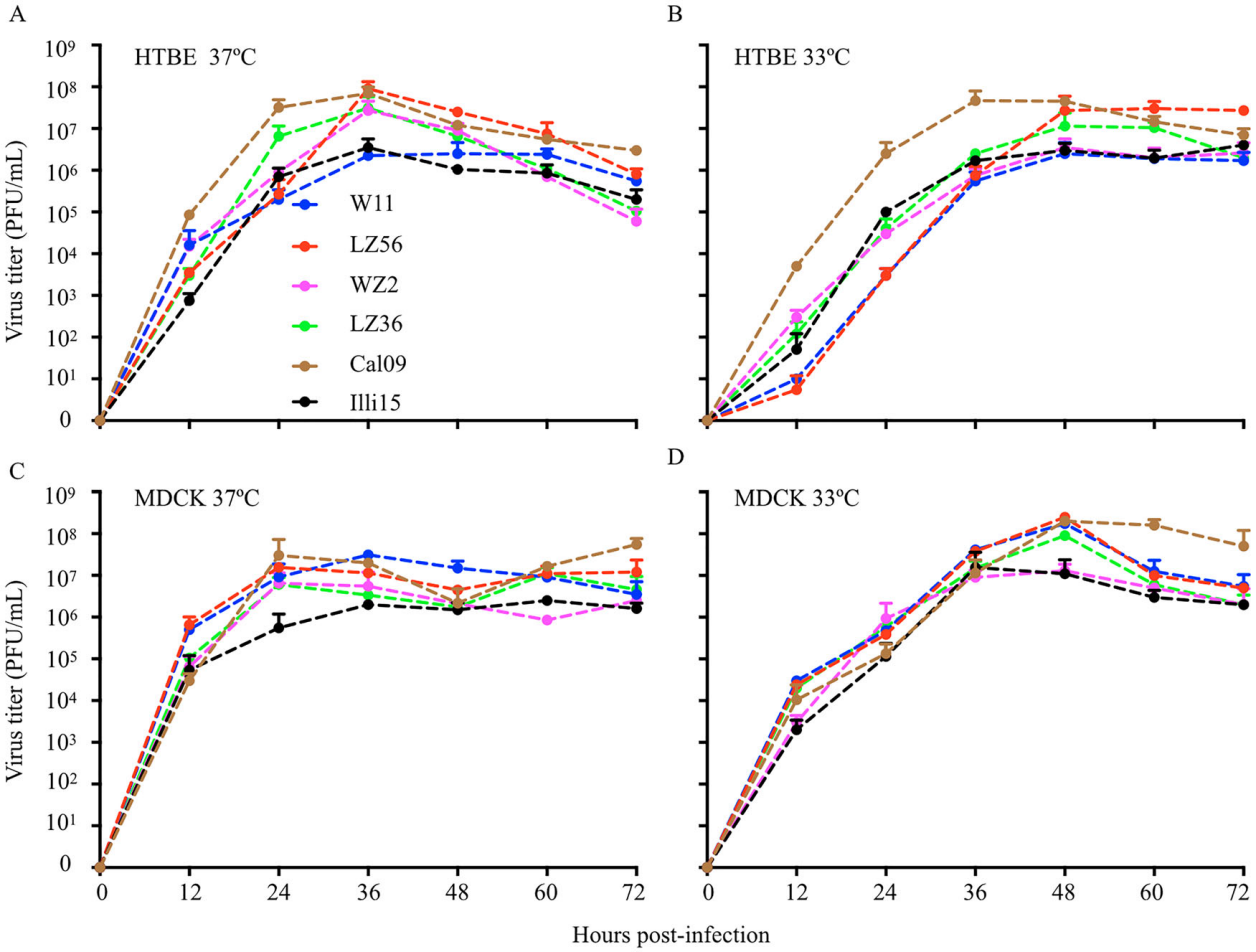
Growth properties of H1N1 IAVs-C in differentiated HTBE cells (A & B) and MDCK cells (C & D). Cells were infected with viruses at MOI of 0.01 and incubated at 37°C (A & C) and 33°C (B & D). Supernatants were harvested at the indicated time points and the virus titers were determined by means of plaque assays in MDCK cells. Error bars indicated SDs from three independent experiments. HTBE, human tracheobronchial epithelial; MDCK, main Darby canine kidney; MOI, multiplicity of infection.

Efficient replication is thought to be essential for viral transmission among hosts. To characterize the replicative ability of H1N1 IAVs-C in both human and canine cells, we infected differentiated HTBE cells and MDCK cells at an MOI of 0.01 at 33°C and 37°C, temperatures corresponding to the temperatures of the upper and lower respiratory tract of humans, respectively (Figure 6). In both canine MDCK and human HTBE cells, all the viruses replicated efficiently. Compared to 37°C, viruses replicated slowly at the early points and reached its highest titers at later time points at 33°C (Figure 6). These results demonstrate that H1N1 IAVs-C replicate efficiently in both human and canine cells.

## Discussion

Our study shows that the swine-origin H1N1 IAVs-C isolated in 2013–2015 replicated efficiently in the respiratory tracts of mice and guinea pigs and that three swine-origin H1N1 IAVs-C were lethal in mice and more pathogenic than the pandemic 2009/H1N1 virus. Notably, three out of four H1N1 IAVs-C viruses were transmissible in guinea pigs via respiratory droplets. Moreover, the H1N1 IAVs-C replicated efficiently in the differentiated HTBE cells. Human infections with the same genotypes of swine-origin H1N1 IAVs-C have been reported, which suggest that these viruses could pose a risk to humans. It will be important to continue monitoring for the presence of these viruses in the canine population and to determine whether they are still evolving and changing genetically and phenotypically.

The pathogenicity and transmission of IAVs are polygenic traits. They can be enhanced either by individual gene mutations [4,5,42] or by genome reassortments [32]. Previous studies showed that the prototypical Eurasian H1N1 swine-origin IAVs (IAVs-S) were non-lethal in mice [43], and that the H1N1/2009 pandemic internal genes, especially PA and M genes, could elevate the virulence and transmission of reassortant viruses [32,33,44]. All of our swine-origin H1N1 IAVs-C were lethal in mice. Notably, the most virulent virus (LZ56) contains a glycine at position 225 in the HA gene, which is a potential virulence marker in mammals [45]. However, the reassortant IAVs-C (LZ36) containing the NS gene of canine H3N2, was avirulent in mice. We also performed transmission studies in guinea pigs and detected H1N1 IAVs-C transmission via direct contact in all the groups. We further evaluated the aerosol transmission of H1N1 IAVs-C and found that three out of the four H1N1 IAVs-C were transmissible via respiratory droplets. Similar to the pathogenicity studies, the transmission efficiency was also different between swine-origin H1N1 IAVs-C and the reassortant H1N1 IAV-C containing the NS gene of canine H3N2 virus origin. This might be due to differences in NS1 functionality. However, since amino acid differences exist in all the proteins of these four H1N1 IAVs-C (Table S1), it remains to be seen which gene or mutation contributed to the different phenotype of virulence and transmission of these viruses.

The preference for human-like receptor-binding is a crucial determinant for an influenza virus highly transmissible in humans [4]. Dogs as an influenza virus reservoir have acquired H3N8 and H3N2 viruses, which have been shown to bind preferentially to SA*α*2,3 receptors (Figure 1 and previous studies [46,47]). However, our results demonstrated that H1N1 IAVs-C possess strong binding affinity to the human-type receptors, with only low-to-moderate binding ability to avian-type receptors. These results suggest that H1N1 IAVs-C have fewer natural barriers than H3N8 or H3N2 IAVs-C to become established in humans.

Vaccination coverage among adults during the 2017–18 season in the United States was only 37.1% [48]. In our study, even though, 80.1% of the donors’ HI titer were equal or greater than 40 against the H1N1/2009 pandemic human influenza A virus, the cross-reactive antibody response against H1N1 IAVs-C was quite limited. Besides, a previous study has shown that a two-dose vaccination regimen with seasonal trivalent inactivated influenza vaccine could not substantially improve the level of cross-reactive antibodies towards Eurasian H1N1 viruses [49]. Therefore, preexisting immunity in humans may not be sufficient to prevent H1N1 IAVs-C infections.

Our previous study demonstrated that swine-origin H1N1 IAVs have emerged in canines and that dogs served as “mixing vessels”, facilitating reassortment with other canine influenza viruses, such as H3N2s [13]. With the expansion of the IAVs-C gene pool and extensive contacts between humans and pets, the potential threat posed by IAVs-C is also increased. The Centers for Disease Control and Prevention in the United States developedan Influenza Risk Assessment Tool (IRAT), which was created in collaboration with an international group of influenza experts to evaluate influ­enza viruses that are not yet readily transmissible in human population for their potential risk of emergence and poten­tial effect on the public’s health [50]. By using IRAT, we evaluated the pandemic potential of different influenza A virus strains. Similar to the newly emerged highly pathogenic avian influenza H7N9, we found that our H1N1 IAVs-C were also categorized into a moderate-to-high level in potential risk for emergence in humans (Table S4) with a moderate-to-high potential public health impact (Table S5). To our knowledge, H1N1 IAVs-C are the only viruses circulating in a pet population that have phenotypes conducive to human transmission. However, at this time there is no surveillance (serological or virological) for these viruses in humans in Southern China, and the frequency of human exposure or possible limited human transmission remains unknown. Therefore, further surveillance is needed for these canine influenza viruses and their reassortants in multiple mammalian hosts.

## Supplementary Material

Supplemental Material
