# Supplementary material for: Characterization of swine-origin H1N1 canine influenza viruses
Source: Emerg Microbes Infect. 2019 Jul 9;8(1):1017–26. doi: 10.1080/22221751.2019.1637284 (PMC7011970; doi:10.1080/22221751.2019.1637284)
Supplement: Supplemental Material [file TEMI_A_1637284_SM4209.zip › IAV_C_Technical_Appendix Final.docx]

**Characterization of swine-origin H1N1 canine influenza viruses**

**Technical Appendixa**

**Materials and methods**

**Viruses.** Influenza A/canine/Guangxi/WZ11/2013 (H1N1) [WZ11], A/canine/Guangxi/LZ56/2015 (H1N1) [LZ56], A/canine/Guangxi/WZ2/2013 (H1N1) [WZ2], and A/canine/Guangxi/LZ36/2015 (H1N1) [LZ36] viruses were isolated from swabs collected from dogs with influenza-like symptoms in Southern China [1]. Influenza A/Bethesda/P0055/2015 (H3N2) [Beth15] provided by the Centers of Excellence for Influenza Research and Surveillance (CEIRS), A/California/04/2009 (H1N1) [Cal09] [2], A/canine/Illinois/41915/2015 (H3N2) [Illi15] provided by CEIRS [3], A/New York/08-1326/2008 (H1N1) [NY08] provided by Kris White [4], and a low pathogenic version (HALO) A/Viet Nam/1203/2004 (H5N1) [Viet04] [5] viruses were used as controls. All viruses were propagated in MDCK cells.

H7Nx reassortant IAV viruses were generated by reverse genetics as described previously [6]. These viruses contain the HA (H7) gene from influenza A/Hunan/02285/2017 (H7N9) virus, gene segments encoding internal proteins from influenza A/Puerto Rico/8/1934 virus, and one of the following NA gene segments: N1 of WZ11, LZ56, WZ2, LZ36, or Cal09 IAVs; and N2 of A/canine/Indiana/1177/-17-1/2017 (H3N2) [Indi17] provided by CERIS. H7Nx viruses were propagated in 10-day-old specific-pathogen-free (SPF) embryonated chicken eggs at 37°C for two days.

**Cells.** Human embryonic kidney 293T cells and Madin-Darby Canine Kidney (MDCK) cells were maintained in Dulbecco's Modification of Eagle's Medium (DMEM; Corning) supplemented with 10% fetal bovine serum (Peak Serum).

Human tracheobronchial epithelial cells (HTBE) were obtained from Lonza. The HTBE cells monolayers were cultured and differentiated according to previously published methods with minor modification [7]. Briefly, cells were seeded on 12-mm Transwell filters in 12-well plate and incubated in primary human airway epithelial cell expansion medium (PneumaCult™-Ex Plus Medium; STEMCELL). For differentiation, cells were cultured in an air-liquid interface for 4-6 weeks. Medium in the lower compartment was renewed every 24 h.

**Flow cytometry-based binding assay.** Receptor specificity was analyzed by use of flow cytometry-based binding assay as previously described [7]. Five different biotinylated glycans, containing α-2,6- linked sialic acids (SAα2,6) or α-2,3- linked sialic acids (SAα2,3) and conjugated with a polyacrylamide (PAA) support, were included in the assay: 6'SLN [Neu5Acα2,6Galβ1-4GlcNAcβ-PAA]; 6’sDi-LN [Neu5Acα2,6(Galβ1 -4GlcNAc)2β-PAA)]; 3′SLN [Neu5Acα2,3Galβ1-4GlcNAcβ-PAA]; 3′sDi-LN [Neu5Acα2,3(Galβ1-4GlcNAc)2β-PAA]; and 3′sTri-LN [Neu5Acα2,3 (Galβ1-4GlcNAc)3β-PAA)]. 6'SLN, 6’sDi-LN, 3′sDi-LN and 3′sTri-LN were kindly provided by the Consortium for Functional Glycomics. 3′SLN was obtained from GlycoTech Corporation, USA. A scheme of the composition of the glycans is shown in Figure S1A.

The experimental procedure of testing receptor-binding specificity is shown in Figure S1B. MDCK cells were infected at a multiplicity of infection (MOI) of 5 for 24 h with the corresponding IAV viruses. Next, cells were harvested, washed three times with cold phosphate-buffered saline (PBS), and then incubated with 10 µg/mL of each isoform of biotinylated glycan and anti-M2 antibody E10 (Mount Sinai Hybridoma Shared Research Facility). Then cells were washed with PBS-1% bovine serum albumin (BSA) and incubated with streptavidin-fluorescein isothiocyanate (FITC) and Alexa Flour® 647 tagged secondary anti-mouse antibody (Life Technologies). Oseltamivir (1 μmol/L) was used in both incubations to block the NA activity. Flow cytometry was performed and analyzed with FlowJo software. Example of gating and analyzing strategy were shown in Figure S1 C, D, and E.

**Animals.** All animal procedures were performed following protocols approved by the Icahn School of Medicine at Mount Sinai Institutional Animal Care and Use Committee (IACUC). All the animal studies were carried out in strict accordance with the recommendations in the Guide for the Care and Use of Laboratory Animals of the National Research Council.

Six- to eight-week-old female BALB/c mice were obtained from Jackson Laboratories (Bar Harbor, ME). Mice were anesthetized by intraperitoneal injection of a mixture of ketamine and xylazine before infection.

Five- to six-week-old female Hartley strain guinea pigs weighing 350 to 400 g were obtained from Charles River Laboratories (Kingston, NY). Guinea pigs were anesthetized by intramuscular administration of ketamine and xylazine for all of the procedures described here, including infection, nasal washes, and bleeding.

**Mouse studies.** Groups of six mice were intranasally (i.n.) infected with 10^6^ plaque-forming unite (PFU) of viruses in a volume of 50 μL, three mice were euthanized on 3 and 5 days post-inoculation (d.p.i.), respectively. The lung and nasal turbinates were collected to determine virus titers by plaque assay on MDCK cells.

Groups of five mice were inoculated i.n. with 10^2^, 10^3^, 10^4^, 10^5^, or 10^6^ PFU of virus. Mice were monitored daily for clinical signs of illness and weight loss. Upon reaching 75% of initial body weight, animals were humanely euthanized with carbon dioxide (CO_2_) as per the IACUC protocol.

**Guinea pig studies.** To investigate the transmissibility of IAVs-C, groups of three animals were inoculated i.n. with 10^6^ PFU of virus in a 300 μL volume per guinea pig (150 μL per nostril) and housed in a cage placed inside an isolator.

For direct transmission, three naïve animals were introduced into the same cage at 24 hours post-infection (h.p.i.) later [8]. For the airborne transmission, three naïve animals were placed in an adjacent cage (4 cm away) at 24 h.p.i..As previously described [4, 9], the cages of virus-inoculated and virus-exposed guinea pig were aligned in pairs so that the wire mesh sides were directly opposed, allowing air to flow freely between cages but precluding direct contact between animals. The ambient conditions were set at 20°C -22°C and 30%-40% relative humidity.

Nasal washes were collected at 2-day intervals, beginning on two d.p.i. (one day post-exposure) and titrated on MDCK cells. Sera were collected from all animals on 15 d.p.i. and treated with Vibrio cholerae (Denka-Seiken) receptor-destroying enzyme before being tested for the presence of hemagglutination inhibiting (HI) antibody with 0.5% (vol/vol) turkey red blood cells.

**Phylogenetic analysis.** To capture the entire multi-host phylogenetic relationships of H1 HA and N1 NA genes of IAVs detected so far in the world, 20,153 full-length H1 HAs and 22,647 full-length N1 NAs from canines, swine, humans, birds, and other species were downloaded from the Influenza Virus Resource at NCBI’s GenBank. Sequences were aligned using MAFFT. Alignments were viewed, edited, and translated into amino acid sequences using AliView[10]. Initial phylogenetic trees were constructed using the neighbor-joining method available in MAFFT. From initial trees, the final set of H1 HA (194) and N1 NA (177) representative sequences were selected to identify interspecies transmission events of H1 and N1 IAVs from birds, swine, dogs, and humans. All the trees were visualized by forester [11].

**Human plasma samples.** We collected plasma from 36 study participants free of acute diseases (defined as “healthy”). Blood samples were obtained between November 2016 and December 2018. Specifically, 17 samples were collected in 2016, 16 in 2017 and 3 in 2018. All the study participants are representatives of the general urban population found in North American metropolitan cities. We have no information on how often individual participants interact with domesticated or wild dogs. Informed consent was obtained prior to study participation. Plasma samples were stored at -80°C until use. 220 μL human plasma was heat treated at 56°C for 30 minutes. Samples were then treated for use in hemagglutination inhibition (HI) assay and neuraminidase inhibition (NI) assay. Table S2 summarizes the demographics of the donors.

**HI assay.** The HI assay was conducted as described elsewhere [12]. In brief, working stocks of each IAV virus strain were prepared by diluting the virus stock to a final HA titer of 8 HA units/50 µL. Serial 2-fold dilutions of treated serum (25 µL per well) were incubated in duplicate with 25 µL per well of working stock of each virus for 30-min at room temperature. To each well, 50 µL of a 0.5% suspension of turkey red blood cells was added and incubated for 45-min incubation at 4°C. The HI titer was obtained by multiplying by 10 the reciprocal dilution of the last reacting well.

**NI assay.** To determine the ideal stock virus concentration be used in the NI assay, an enzyme-linked lectin assay (ELLA) was first performed for all reassortant H7Nx viruses, as previously described, [13, 14]. Briefly, ELISA 96 well plates were coated with fetuin. 100 µL of serial 2-fold virus dilutions were added to the fetuin coated plates. The plates were incubated for two hat 37°C and washed four times with PBS supplemented with 0.1% Tween 20 (PBS-T, Sigma). Peanut agglutinin conjugated to horseradish peroxidase (PNA-HRP, Sigma) at a concentration of 5 µg/mL diluted in PBS was added to the plate (100 µL per well) and incubated in the dark for 1.5 h. The plates were washed four times with PBS-T, and 100 µL SigmaFast OPD (Sigma) was added. After 5 minutes the reaction was stopped by the addition of 50 µL 3 mol/L hydrochloric acid (HCl, Thermo Fisher). The plates were read at the absorption of 490 nm using a Synergy H1 hybrid multimode microplate reader (BioTek). The data were analyzed in Microsoft Excel and GraphPad Prism 7. The data were fitted to a nonlinear regression curve to determine the effective concentration 50 (EC_50_) of the different viruses. Working stocks of each H7Nx virus were prepared by diluting the virus stock to two times the EC_50_.

For the NI assay, 75 µL of treated human plasma was serially diluted two-fold. Two times the EC_50_ virus (75 µL) was added to each well and incubated for 1.5 h at room temperature on a shaker. Hundred µL of the virus/plasma mixture was added to the fetuin coated plates and incubated at 37°C for two h. The plates were washed four times with PBS-T, 100 µL of PNA-HRP was added and incubated for 1.5 h at room temperature in the dark. The plates were washed four times with PBS-T and developed as described above. The absorption was read and the data analyzed in Microsoft Excel and GraphPad Prism 7.

The inhibition of the neuraminidase activity was calculated by dividing the values obtained from the plate reader by the average of the values for the virus-only control wells and then multiplying by 100. The result of this calculation was subtracted from 100 to determine percent inhibition. These data were fitted to a nonlinear regression curve to calculate the NI titers in human sera as previously described [15].

**Viral sensitivity to M2 and NA inhibitors in vitro.** Plaque reduction assays were performed to test the viral sensitivity to M2 and NA inhibitors according to previous reports [16, 17]. Viruses were used to infect MDCK cells in the presence or absence of compounds to evaluate their antiviral activity. Briefly, a confluent monolayer of MDCK cells (6-well plates) was incubated with ~100 PFU/200 µL in Opti-MEM (Gibco) at 37 ºC for one h. The virus inoculums were removed, and the cells were washed twice with PBS. The cells were then overlaid with DMEM containing 1.2% Avicel (FMC BioPolymer) and tosyl phenylalanyl chloromethyl ketone (TPCK)-treated trypsin (1.0 mg/mL). To examine the effect of the compounds on plaque formation, the overlay media were supplemented with Amantadine (Sigma) or Oseltamivir carboxylate (Roche) provided by Megan L. Shaw at testing concentrations. After an incubation of 48 h at 37 ºC, the monolayers were fixed and stained with crystal violet dye solution (0.2% crystal violet, 20% methanol). Plaques were counted, and the fifty percent inhibitory concentration (IC_50_) values were subsequently determined by GraphPad Prism 7.

**Growth kinetics of Viruses in Cell Culture.** Differentiated HTBE cells were infected with viruses at a multiplicity of infection (MOI) of 0.01, incubated for one h at 37 °C, washed twice, and subsequently cultured with PneumaCult™-Ex Plus Medium in the lower compartment and incubated at either 33 °C or 37 °C. Viral supernatants were collected at different times post-infection by adding 200 µL of PBS to each well. Cells were incubated for 30-min, viral supernatants were collected and titrated by plaque assay.

MDCK cells were infected with viruses at an MOI of 0.01, incubated for one h at 37 °C, washed twice, and then cultured with Opti-MEM and TPCK-treated trypsin at 33°C and 37°C for 72 h. Virus titers were determined at the indicated time points.

**Statistical analysis.**GraphPad Prism 7 was used to perform all statistical analyses. The HI and NI titers of human serum between different viruses were statistically analyzed by One-way analysis of variance (ANOVA).

**References:**

1. Chen Y, Trovao NS, Wang G, Zhao W, He P, Zhou H, et al. Emergence and Evolution of Novel Reassortant Influenza A Viruses in Canines in Southern China. MBio. 2018;9(3).
2. Martinez-Romero C, de Vries E, Belicha-Villanueva A, Mena I, Tscherne DM, Gillespie VL, et al. Substitutions T200A and E227A in the hemagglutinin of pandemic 2009 influenza A virus increase lethality but decrease transmission. J Virol. 2013;87(11):6507-11.
3. Rodriguez L, Nogales A, Reilly EC, Topham DJ, Murcia PR, Parrish CR, et al. A live-attenuated influenza vaccine for H3N2 canine influenza virus. Virology. 2017;504:96-106.
4. Bouvier NM, Rahmat S, Pica N. Enhanced mammalian transmissibility of seasonal influenza A/H1N1 viruses encoding an oseltamivir-resistant neuraminidase. J Virol. 2012;86(13):7268-79.
5. Steel J, Lowen AC, Pena L, Angel M, Solorzano A, Albrecht R, et al. Live attenuated influenza viruses containing NS1 truncations as vaccine candidates against H5N1 highly pathogenic avian influenza. J Virol. 2009;83(4):1742-53.
6. Fodor E, Devenish L, Engelhardt OG, Palese P, Brownlee GG, Garcia-Sastre A. Rescue of influenza A virus from recombinant DNA. J Virol. 1999;73(11):9679-82.
7. Ramos I, Bernal-Rubio D, Durham N, Belicha-Villanueva A, Lowen AC, Steel J, et al. Effects of receptor binding specificity of avian influenza virus on the human innate immune response. J Virol. 2011;85(9):4421-31.
8. Wang G, Deng G, Shi J, Luo W, Zhang G, Zhang Q, et al. H6 influenza viruses pose a potential threat to human health. J Virol. 2014;88(8):3953-64.
9. Lowen AC, Mubareka S, Tumpey TM, Garcia-Sastre A, Palese P. The guinea pig as a transmission model for human influenza viruses. Proc Natl AcadSci U S A. 2006;103(26):9988-92.
10. Larsson A. AliView: a fast and lightweight alignment viewer and editor for large datasets. Bioinformatics. 2014;30(22):3276-8.
11. masek CM, Eddy SR. ATV: display and manipulation of annotated phylogenetic trees. Bioinformatics. 2001;17(4):383-4.
12. Seibert CW, Rahmat S, Krause JC, Eggink D, Albrecht RA, Goff PH, et al. Recombinant IgA is sufficient to prevent influenza virus transmission in guinea pigs. J Virol. 2013;87(14):7793-804.
13. Rajendran M, Nachbagauer R, ErmLer ME, Bunduc P, Amanat F, Izikson R, et al. Analysis of Anti-Influenza Virus Neuraminidase Antibodies in Children, Adults, and the Elderly by ELISA and Enzyme Inhibition: Evidence for Original Antigenic Sin. MBio. 2017;8(2).
14. Krammer F, Fouchier RAM, Eichelberger MC, Webby RJ, Shaw-Saliba K, Wan H, et al. NAction! How Can Neuraminidase-Based Immunity Contribute to Better Influenza Virus Vaccines? MBio. 2018;9(2).
15. Couzens L, Gao J, Westgeest K, Sandbulte M, Lugovtsev V, Fouchier R, et al. An optimized enzyme-linked lectin assay to measure influenza A virus neuraminidase inhibition antibody titers in human sera. J Virol Methods. 2014;210:7-14.
16. Matrosovich M, Matrosovich T, Garten W, Klenk HD. New low-viscosity overlay medium for viral plaque assays. Virol J. 2006;3:63.
17. Hu Y, Musharrafieh R, Ma C, Zhang J, Smee DF, DeGrado WF, et al. An M2-V27A channel blocker demonstrates potent in vitro and in vivo antiviral activities against amantadine-sensitive and -resistant influenza A viruses. Antiviral Res. 2017;140:45-54.

Table S1. Amino acid differences among H1N1 IAVs-C viruses used in this study**^#^**

| Viral protein | Amino acid positions | Virus | | | | Viral protein | Amino acid positions | Virus | | | |
| --- | --- | --- | --- | --- | --- | --- | --- | --- | --- | --- | --- |
|  |  | WZ11 | LZ56 | WZ2 | LZ36 |  |  | WZ11 | LZ56 | WZ2 | LZ36 |
| PB2 | 299 | **R** | **R** | **R** | K | NS1 | 44 | **K** | **K** | **K** | R |
|  | 398 | **I** | M | **I** | **I** |  | 53 | A | A | D | D |
|  | 509 | R | **G** | **G** | **G** |  | 57 | **A** | **A** | **A** | T |
|  | 510 | E | **N** | **N** | **N** |  | 59 | **L** | **L** | **L** | R |
|  | 511 | R | **V** | **V** | **V** |  | 60 | **V** | **V** | **V** | I |
|  | 512 | T | **L** | **L** | **L** |  | 70 | **K** | **K** | **K** | E |
|  | 513 | I | **L** | **L** | **L** |  | 74 | **S** | **S** | **S** | D |
|  | 514 | V | **S** | **S** | **S** |  | 75 | **E** | **E** | **E** | K |
|  | 515 | S | **P** | **P** | **P** |  | 76 | **T** | **T** | **T** | A |
|  | 516 | R | **E** | **E** | **E** |  | 81 | T | **I** | **I** | **I** |
|  | 517 | R | **E** | **E** | **E** |  | 86 | **T** | **T** | **T** | A |
|  | 715 | **N** | **N** | **N** | S |  | 90 | **I** | **I** | **I** | L |
|  | 731 | I | **V** | **V** | **V** |  | 91 | **A** | **A** | **A** | T |
|  | 740 | **D** | **D** | N | **D** |  | 106 | V | **M** | **M** | **M** |
| PB1 | 195 | **M** | **M** | **M** | V |  | 108 | R | R | K | K |
|  | 365 | **R** | **R** | **R** | P |  | 109 | **H** | **H** | **H** | Q |
|  | 645 | **V** | **V** | **V** | I |  | 111 | **I** | **I** | **I** | V |
| PA | 71 | **L** | **L** | P | **L** |  | 112 | **V** | **I** | **V** | A |
|  | 99 | **G** | **G** | R | **G** |  | 117 | **V** | **V** | **V** | I |
|  | 129 | V | V | I | I |  | 118 | **R** | **R** | **R** | K |
|  | 225 | **S** | **S** | N | **S** |  | 124 | **I** | **I** | **I** | M |
|  | 269 | **R** | **R** | **R** | K |  | 125 | **E** | **E** | **E** | N |
|  | 356 | **K** | **K** | R | **K** |  | 129 | **I** | **I** | **I** | T |
|  | 401 | **R** | **R** | **R** | K |  | 139 | **N** | **N** | **N** | E |
|  | 648 | **S** | **S** | N | **S** |  | 140 | **R** | **R** | Q | **R** |
|  | 716 | E | E | K | K |  | 145 | **V** | **V** | **V** | I |
| HA1* | 4 | I | I | R | R |  | 152 | **E** | **E** | **E** | N |
|  | 11 | **A** | **A** | **A** | V |  | 153 | **G** | **G** | **G** | E |
|  | 61 | **L** | **L** | V | **L** |  | 166 | **L** | F | **L** | **L** |
|  | 65 | **I** | **I** | T | **I** |  | 171 | **D** | **D** | N | **D** |
|  | 86 | **S** | **S** | L | **S** |  | 172 | **E** | **E** | **E** | K |
|  | 113 | **A** | **A** | **A** | T |  | 178 | **V** | **V** | **V** | I |
|  | 136 | N | N | K | K |  | 189 | **S** | **S** | **S** | D |
|  | 239 | **E** | G | **E** | **E** |  | 192 | **V** | **V** | **V** | I |
|  | 275 | **K** | **K** | Q | E |  | 197 | **S** | **S** | **S** | N |
|  | 279 | **S** | **S** | **S** | N |  | 205 | **K** | R | **K** | S |
|  | 319 | E | E | K | K |  | 206 | **H** | **H** | **H** | S |
| HA2 | 61 | **I** | **I** | **I** | T |  | 207 | **N** | **N** | **N** | D |
|  | 67 | **G** | **G** | **G** | S |  | 209 | **D** | **D** | **D** | N |
|  | 128 | **N** | **N** | **N** | S |  | 216 | **S** | **S** | **S** | P |
|  | 158 | **Q** | H | **Q** | **Q** |  | 217 | **E** | **E** | **E** | K |
| NP | 107 | E | **D** | **D** | **D** |  | 220 | **X** | **X** | **X** | R |
|  | 223 | **V** | **V** | **V** | I |  | 221 | - | - | - | K |
|  | 322 | **M** | V | **M** | **M** |  | 222 | - | - | - | M |
|  | 458 | **K** | **K** | Q | **K** |  | 223 | - | - | - | A |
|  | 504 | N | **S** | **S** | **S** |  | 224 | - | - | - | R |
| NA | 52 | **N** | **N** | **N** | T |  | 225 | - | - | - | T |
|  | 67 | I | **V** | **V** | **V** |  | 226 | - | - | - | I |
|  | 173 | **R** | **R** | K | **R** |  | 227 | - | - | - | E |
|  | 255 | **V** | **V** | I | **V** |  | 228 | - | - | - | S |
|  | 287 | **K** | **K** | E | **K** |  | 229 | - | - | - | E |
|  | 289 | **T** | **T** | I | **T** |  | 230 | - | - | - | I |
|  | 322 | **F** | **F** | L | **F** |  | 231 | - | - | - | X |
|  | 340 | **S** | **S** | P | **S** | NEP | 3 | **S** | **S** | F | **S** |
|  | 461 | V | **A** | **A** | **A** |  | 14 | **M** | **M** | **M** | I |
| M1 | 30 | **S** | **S** | G | **S** |  | 31 | **I** | **I** | **I** | M |
|  | 36 | **N** | **N** | S | **N** |  | 32 | **V** | **V** | **V** | I |
|  | 126 | G | G | S | S |  | 34 | **R** | **R** | **R** | Q |
|  | 248 | **M** | **M** | I | **M** |  | 40 | **V** | **V** | **V** | I |
| M2 | 13 | **S** | **S** | N | **S** |  | 48 | **S** | G | **S** | A |
|  | 14 | **E** | **E** | G | **E** |  | 49 | **I** | **I** | **I** | V |
|  | 19 | **C** | **C** | Y | **C** |  | 52 | **T** | **T** | **T** | M |
|  | 43 | **T** | **T** | L | **T** |  | 57 | **Y** | **Y** | **Y** | S |
|  | 55 | **F** | **F** | L | **F** |  | 60 | **N** | **N** | **N** | S |
| NS1 | 3 | **S** | **S** | F | **S** |  | 63 | **E** | **E** | **E** | G |
|  | 15 | **I** | **I** | **I** | L |  | 70 | **G** | **G** | **G** | S |
|  | 25 | **N** | **N** | **N** | Q |  | 89 | **A** | **A** | **A** | I |

**^#^** Consensus sequences among the four H1N1 IAVs-C are shown in bold; the Receptor-binding domain in HA is shown in red; *, H1 numbering; -, not available.

Amino acid: A, alanine; C, cysteine; D, aspartic acid; E, glutamic acid; F, phenylalanine; G, glycine; H, histidine; I, isoleucine; K, lysine; L, leucine; M, methionine; N, asparagine; P, proline; Q, glutamine; R, arginine; S, serine; T, threonine; V, valine; X, stop; Y, tyrosine.

Table S2. Seroconversion of guinea pig inoculated with or exposed to the H1N1 IAVs-C

| Virus | Seroconversion (HI titer) of direct contact | |  | Seroconversion (HI titer) of respiratory droplets | |
| --- | --- | --- | --- | --- | --- |
|  | Inoculated | Exposed |  | Inoculated | Exposed |
| WZ11 | 3/3(320, 80,640) | 3/3(320, 80, 640) |  | 3/3(160, 320, 160) | 1/3(320, 0, 0) |
| LZ56 | 3/3(320, 160, 160) | 3/3(160, 80, 160) |  | 3/3(640,640,320) | 1/3(0, 0, 640) |
| WZ2 | 3/3(1280, 640, 160) | 3/3(640, 80, 80) |  | 3/3(160, 320, 320) | 1/3(40, 0, 0) |
| LZ36 | 3/3(640, 320, 160) | 3/3(160, 320, 80) |  | 3/3(320, 80, 160) | 0/3(0, 0, 0) |
| Cal09 | ND | ND |  | 3/3(1280, 640, 2560) | 3/3(640, 640.320) |
| Illi15 | 3/3(80, 160, 80) | 1/3(40, 0, 0) |  | ND | ND |

ND, not done.

Table S3. Demographics of human donors

| Gender |  | Number (N=36) |
| --- | --- | --- |
| Female |  | 14 |
| Male |  | 22 |
|  |  |  |
| **Age (years)** |  | **Number (N=36)** |
| 18-59 |  | 31 |
| >60 |  | 5 |
|  |  |  |
| **Ancestry** |  | **Number (N=36)** |
| Caucasian |  | 24 |
| Other |  | 12 |

Table S4. Estimated risk scores of emergence

| Risk element | Weight (W) |  | Avian H1N1 | |  | Avian H7N9 | |  | Human H5N1 | |  | Canine H3N8 | |  | Canine H3N2 | |  | Canine H1N1 | | |
| --- | --- | --- | --- | --- | --- | --- | --- | --- | --- | --- | --- | --- | --- | --- | --- | --- | --- | --- | --- | --- |
|  |  |  | RS | W x RS |  | RS | W x RS |  | RS | W x RS |  | RS | W x RS |  | RS | W x RS |  | RS | W x RS |  |
| Human infections | 0.2929 |  | 2.33 | 0.68 |  | 6.50 | 1.90 |  | 5.67 | 1.66 |  | 1.23 | 0.36 |  | 1.20 | 0.35 |  | 5.44 | 1.59 |  |
| Transmission in lab animals | 0.1929 |  | 2.00 | 0.39 |  | 8.00 | 1.54 |  | 2.50 | 0.48 |  | 4.50 | 0.87 |  | 4.20 | 0.81 |  | 8.00 | 1.54 |  |
| Receptor binding | 0.1429 |  | 2.00 | 0.29 |  | 8.00 | 1.14 |  | 3.30 | 0.47 |  | 2.20 | 0.31 |  | 2.42 | 0.35 |  | 8.70 | 1.24 |  |
| Population immunity | 0.1096 |  | 3.00 | 0.33 |  | 8.75 | 0.96 |  | 8.67 | 0.95 |  | 7.47 | 0.82 |  | 7.50 | 0.82 |  | 7.60 | 0.83 |  |
| Infections in animals | 0.0846 |  | 2.00 | 0.17 |  | 6.40 | 0.54 |  | 7.25 | 0.61 |  | 7.50 | 0.63 |  | 7.22 | 0.61 |  | 9.00 | 0.76 |  |
| Genomic variation | 0.0646 |  | 3.00 | 0.19 |  | 7.70 | 0.50 |  | 4.00 | 0.26 |  | 3.33 | 0.22 |  | 3.40 | 0.22 |  | 8.00 | 0.52 |  |
| Antigenic relatedness | 0.0479 |  | 2.00 | 0.10 |  | 5.25 | 0.25 |  | 6.00 | 0.29 |  | 5.40 | 0.26 |  | 4.67 | 0.22 |  | 5.00 | 0.24 |  |
| Global distribution in animals | 0.0336 |  | 2.50 | 0.08 |  | 5.40 | 0.18 |  | 5.50 | 0.18 |  | 5.22 | 0.18 |  | 6.00 | 0.20 |  | 7.60 | 0.26 |  |
| Disease severity & pathogenesis | 0.0211 |  | 2.25 | 0.05 |  | 8.50 | 0.18 |  | 8.50 | 0.18 |  | 2.25 | 0.05 |  | 2.79 | 0.06 |  | 8.00 | 0.17 |  |
| Antiviral and treatment options | 0.001 |  | 2.25 | 0.00 |  | 6.50 | 0.01 |  | 4.50 | 0.00 |  | 2.33 | 0.00 |  | 2.00 | 0.00 |  | 5.50 | 0.01 |  |
| Total |  |  |  | 2.28 |  |  | 7.21 |  |  | 5.09 |  |  | 3.69 |  |  | 3.65 |  |  | 7.16 |  |
| Risk range |  |  | Low | |  | Moderate-high | |  | Moderate | |  | Low | |  | Low | |  | Moderate -high | | |

Avian H1N1, A/duck/New York/1996; Avian H7N9, A/Chicken/Guangdong/SD008/2017; Human H5N1, A/Vietnam/1203/2004; Canine H3N8, A/canine/Florida/14/2006; Canine H3N2, A/canine/Illinois/41915/2015; Canine H1N1, A/canine/Guangxi/WZ2/2013; RS, Risk Scores.

Table S5. Estimated potential public health impact

| Risk element |  | Weight (W) |  | Avian H1N1 | |  | Avian H7N9 | |  | Human H5N1 | |  | Canine H3N8 | |  | Canine H3N2 | |  | Canine H1N1 | |
| --- | --- | --- | --- | --- | --- | --- | --- | --- | --- | --- | --- | --- | --- | --- | --- | --- | --- | --- | --- | --- |
|  |  |  |  | RS | W x RS |  | RS | W x RS |  | RS | W x RS |  | RS | W x RS |  | RS | W x RS |  | RS | W x RS |
| Disease severity & pathogenesis |  | 0.2929 |  | 2.25 | 0.66 |  | 8.50 | 2.49 |  | 8.50 | 2.49 |  | 2.25 | 0.66 |  | 2.79 | 0.82 |  | 8.00 | 2.34 |
| Population immunity |  | 0.1929 |  | 3.00 | 0.58 |  | 8.75 | 1.69 |  | 8.67 | 1.67 |  | 7.47 | 1.44 |  | 7.50 | 1.45 |  | 7.60 | 1.47 |
| Human infections |  | 0.1429 |  | 2.33 | 0.33 |  | 6.50 | 0.93 |  | 5.67 | 0.81 |  | 1.23 | 0.18 |  | 1.20 | 0.17 |  | 5.44 | 0.78 |
| Antiviral and treatment options |  | 0.1096 |  | 2.25 | 0.25 |  | 6.50 | 0.71 |  | 4.50 | 0.49 |  | 2.33 | 0.26 |  | 2.00 | 0.22 |  | 5.50 | 0.60 |
| Antigenic relatedness |  | 0.0846 |  | 2.00 | 0.17 |  | 5.25 | 0.44 |  | 6.00 | 0.51 |  | 5.40 | 0.46 |  | 4.67 | 0.40 |  | 5.00 | 0.42 |
| Receptor binding |  | 0.0646 |  | 2.00 | 0.13 |  | 8.00 | 0.52 |  | 3.30 | 0.21 |  | 2.20 | 0.14 |  | 2.42 | 0.16 |  | 8.70 | 0.56 |
| Genomic variation |  | 0.0479 |  | 3.00 | 0.14 |  | 7.70 | 0.37 |  | 4.00 | 0.19 |  | 3.33 | 0.16 |  | 3.40 | 0.16 |  | 8.00 | 0.38 |
| Transmission in lab animals |  | 0.0336 |  | 2.00 | 0.07 |  | 8.00 | 0.27 |  | 2.50 | 0.08 |  | 4.50 | 0.15 |  | 4.20 | 0.14 |  | 8.00 | 0.27 |
| Global distribution in animals |  | 0.0211 |  | 2.50 | 0.05 |  | 5.40 | 0.11 |  | 5.50 | 0.12 |  | 5.22 | 0.11 |  | 6.00 | 0.13 |  | 7.60 | 0.16 |
| Infections in animals |  | 0.0100 |  | 2.00 | 0.02 |  | 6.40 | 0.06 |  | 7.25 | 0.07 |  | 7.50 | 0.08 |  | 7.22 | 0.07 |  | 9.00 | 0.09 |
| Total |  |  |  |  | 2.40 |  |  | 7.60 |  |  | 6.65 |  |  | 3.63 |  |  | 3.71 |  |  | 7.08 |
| Risk range |  |  |  | Low | |  | Moderate-high | |  | Moderate | |  | Low | |  | Low | |  | Moderate-high | |

Avian H1N1, A/duck/New York/1996; Avian H7N9, A/Chicken/Guangdong/SD008/2017; Human H5N1, A/Vietnam/1203/2004; Canine H3N8, A/canine/Florida/14/2006; Canine H3N2, A/canine/Illinois/41915/2015; Canine H1N1, A/canine/Guangxi/WZ2/2013; RS, Risk Scores.

**Figure S1. Details of analyzing receptor-binding specificity of IAV using a flow cytometry-based binding assay.** (A) Structure of Neu5Ac-linked glycan. (B) Experimental procedure of testing receptor-binding specificity. (C) Uninfected cells as control of gating M2 positive cells. (D) M2 positive cells in Viet05 virus-infected cells. (E) Receptor-binding specificity of M2 positive cells gated from (D). M2, matrix protein 2; M2 E10, M2-specific monoclonal antibody recognizing all the viruses used in this study; FSC, forward-scattered light; FITC, fluorescein isothiocyanate.
