# Supplementary figures and images for: Characterization of swine-origin H1N1 canine influenza viruses
Source: Emerg Microbes Infect. 2019 Jul 9;8(1):1017–26. doi: 10.1080/22221751.2019.1637284 (PMC7011970; doi:10.1080/22221751.2019.1637284)

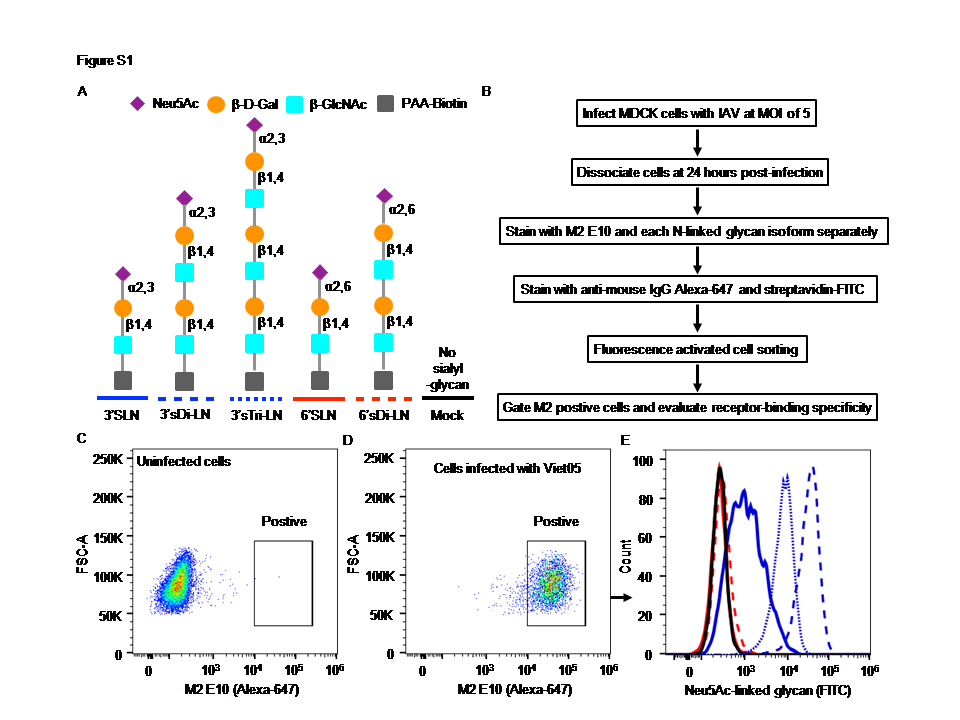

Supplement: Supplemental Material [file TEMI_A_1637284_SM4209.zip › IAV_C_Figures_S1 Final.tif]
